# Understanding Barriers to the Access to Healthcare and Rehabilitation Services: A Qualitative Study with Mothers or Female Caregivers of Children with a Disability in Indonesia

**DOI:** 10.3390/ijerph182111546

**Published:** 2021-11-03

**Authors:** Gregorius Abanit Asa, Nelsensius Klau Fauk, Lillian Mwanri, Paul Russell Ward

**Affiliations:** 1 Sanggar Belajar Alternatif (SALT), Atambua 85711, Indonesia; gorisasa@yahoo.com; 2 College of Medicine and Public Health, Flinders University, P.O. Box 2100, Adelaide 5001, Australia; lillian.mwanri@flinders.edu.au (L.M.); paul.ward@flinders.edu.au (P.R.W.); 3 Institute of Resource Governance and Social Change, Kupang 85221, Indonesia

**Keywords:** children with a disability, mothers or female caregivers, healthcare and rehabilitation services, barriers to accessing services, Belu, Indonesia

## Abstract

Accessibility to healthcare and rehabilitation services for children with a disability (CWD) is essential to improving their health and wellbeing. However, access to the services, especially in many settings in developing countries with scarcity of resources, is still limited. As part of a qualitative study exploring impacts of caring for CWD on mothers or female caregivers and their coping strategies, this paper describes barriers for access to healthcare and rehabilitation services for CWD in Belu district, Indonesia. One-on-one, in-depth interviews were conducted with 22 mothers or female caregivers of CWD. Participants were recruited using a combination of purposive and snowball sampling techniques. These were supplemented with interviews with two staff of disability rehabilitation centers in Belu to understand any additional barriers. Data analysis was guided by a qualitative data analysis framework. Our analysis identified that lack of affordability of healthcare services (high costs and low financial capacity of mothers) was the key barrier for access to healthcare and rehabilitation services CWD. Religious or faith-based factors, such as being a non-Catholic (Belu is predominantly Catholic), converting from Catholic to other religions, and the belief in children’s disability condition as “God’s will”, were also influencing factors for lack of access to the services. Shortage of staff, distrust in the therapy skills of staff at rehabilitation centers, and unavailability of appropriately trained healthcare professionals were structural or system-related barriers. The findings indicate the need for government-owned and run disability rehabilitation centers (not faith-based), the provision of fully subsidised health insurance to provide free services, and the provision of qualified therapists and healthcare professionals (to build trust) in Belu and other similar settings in Indonesia.

## 1. Introduction

The World Health Organization (WHO) estimates that over one billion people across the world live with a disability, and of these, 80% are in developing countries [1]. The report also shows an estimated 90 million children worldwide living with a disability, with 0.7% experiencing severe disability [1]. In Indonesia, there are over 37 million people living with a disability, of which 3.2 million are children [2]. In order to provide optimal quality of life and promote the health and wellbeing of people with a disability, they should have access to appropriate healthcare and rehabilitation services [3]. This paper focuses on understanding the barriers to such services for CWD in Belu, Indonesia.

Evidence suggests that, in many settings with limited resources (such as Indonesia), people with a disability, including children, still experience difficulties in accessing healthcare, rehabilitation, and social support services. For example, the poor financial situation of parents is one of the main barriers to access to services [4,5,6,7,8,9]. Studies in a number of low- and middle-income countries (LMICs) have identified financial barriers, such as the inability of parents to afford the costs for healthcare or treatment services [10,11,12,13], health insurance [14], and/or social protection [9,15]. Social factors, such as stigma and discrimination from community members and healthcare providers towards people with a disability (leading to embarrassment by parents to be “seen” with their child in public), have also been reported as barriers for access to healthcare and rehabilitation services [7,16,17,18,19,20,21]. Lack of social support and discouragement from others to not seek help or support for CWD are also influencing factors for lack of access to appropriate services [20]. Several studies have also reported that limited provision of essential services and rehabilitation centers for CWD are organisational or system-related barriers in LMICs [22,23,24].

Despite a wide range of barriers reported in the aforementioned studies in several LMICs, there is a paucity of evidence in Indonesia, which has over 3 million CWD. In addition, there is a lack evidence on religious or faith-based factors related to both perceived needs for healthcare and rehabilitation services and barriers to the access to the services in the context of Indonesia and globally. This study aims to fill in this gap by exploring barriers to the access of mothers or female caregivers to healthcare and rehabilitation services for their CWD. Belu is predominantly a Catholic area (Catholic (90%), followed by Christian (7%) and Muslim (3%)), with most people actively following religion and holding firmly to religious beliefs and values, which have a significant influence on daily life in shaping their attitudes, behaviours, and how they perceive or respond to issues, such as disability and access to healthcare [25]. The study focuses on exploring the perspectives of mothers and female caregivers due to the reasons that, in Indonesia and many other developing countries, women (e.g., mothers, daughters, and female caregivers), have the responsibility within families to take care of children, husbands, and other household chores [26,27,28,29]. Understanding religious-related barriers and other barriers to the access to healthcare and rehabilitation services is useful to inform disability-related policies and programs designed to help or support the health and wellbeing of CWD.

## 2. Methods

The Consolidated Criteria for Reporting Qualitative Studies (COREQ) checklist for explicit and comprehensive reporting of qualitative study particularly for interviews and focus group discussion (see Figure S1) was used to guide the report of the methods section of this study [30].

### 2.1. Theoretical Framework

The access to healthcare conceptual framework [31] was used to guide the study, including data collection (the interview questions were structured around this framework), data analysis, and discussion of the study findings. The framework suggests that accessibility of healthcare services is determined by a number of factors at individual, structural, and socio-cultural levels. One factor relates to the physical and human resources available in healthcare services, such as the physical existence of both the services at healthcare facilities and appropriately qualified healthcare staff to provide the services. Additional service-related factors relate to the affordability of services for people in healthcare [31,32] and appropriateness of the services in terms of the services meeting the specific needs of the community they serve [31]. On an individual level, potential service users need to actually know about the services, be able to physically (appropriate locations and opening times) and financially access them, and also perceive a need for the services. On a socio-cultural level, the acceptability of the services refers to whether or not the services are provided in a way that is socially, culturally, and religiously acceptable to people [31,33], which includes staff being approachable, being non-discriminatory, and creating a culturally and religiously safe space for all people to access and use. This could also include information about services being provided in appropriate languages and using inclusive and culturally meaningful language [31,33].

### 2.2. Study Setting

Data collection for this paper was conducted in Belu district, East Nusa Tenggara Province, Indonesia. The district has a total population of 204,541 people that are distributed among 12 sub-districts [34]. The district has a state special school for CWD (*Sekolah Luar Biasa Negeri*, also known as SLBN), which provides education for CWD at elementary, junior high school, and senior high school [35,36]. There are also two private rehabilitation centers for CWD, which are called *Pusat Rehabilitasi Hidup Baru* (New Life Rehabilitation Center) and run by sisters of Franciscan, and *Bhakti Luhur,* run by the sisters of ALMA (Asosiasi Lembaga Misionaris Awam) Congregation. The two private rehabilitation centers are part of Catholic mission to help CWD. These rehabilitation centers are inclusive, providing services for all CWD regardless of religious background. Services provided in these rehabilitation centers include physiotherapy, massage therapy, speaking therapy, occupational therapy to help CWD to be independent in daily living, and education. These services are independently provided by these rehabilitation centers as part of the Catholic nuns’ mission to help CWD without any specific collaborations with medical personnel from hospitals in the district. In addition, there is not any specific healthcare service provided in any healthcare facilities in the district for CWD or people living with disability in general. It should be acknowledged that CWD do not fully get special treatment according to their disability needs. The current report shows that the district has 348 CWD, of whom 58 children reside in the two rehabilitation centers, and the remaining 290 children live with parents or caregivers [35,37]. Of the 348 children, only 71 enrolled to the state special school for CWD [35,37].

The population in Indonesia based on the 2020 population census report was 270.20 million people, and about 10% or one out of every 10 Indonesians live in poverty [38]. Indonesian government has implemented a national health care program called the Social Security Administrator for Health (BPJS), providing health insurance for all its citizens [39]. The BPJS consists of *KIS* (The Indonesian Health Card) type, which is fully subsidised by the government or free of charge, and *mandiri* (independent) type, which is partly sudsidised by the government and requires a small amount of monthly insurance fee to be paid by the insurance holders. The amount of the monthly fee depends on the category, which is IDR 35,000 (±$2.5) for third class, IDR 100,000 ($7) for second class, and IDR 150,000 ($10.5) for first class [39]. However, data show that there is a significant number of Indonesians, including people living with a disability, who do not have health insurance, with poor economic condition leading to inability to pay for monthly insurance fee as the main reason [40].

### 2.3. Study Design, Recruitment of Participants, and Data Collection

As part of a qualitative study exploring impacts of caring for CWD on mothers or female caregivers and their coping strategies [35,36], this paper describes barriers to the access to healthcare and rehabilitation services for CWD in Belu district, Indonesia. This study was conducted from August to September 2019. The qualitative design enabled mothers or female caregivers to present and explain their views, understanding, and experiences of trying to access services for their children and to discuss any barriers that they or their children encountered. In this way, we were interested in understanding the reality for our participants, allowing them to talk about the issues that matter to them, which is a key benefit of qualitative research [41,42]. In addition to the interviews with mothers and female caregivers, we also interviewed two staff at rehabilitation services in Belu in order to obtain any additional details about the services offered in Belu for CWD and to triangulate findings from the interviews with mothers or female caregivers.

Twenty-two mothers or female caregivers of CWD and two staff at the rehabilitation centers participated in the study. The recruitment used a combination of purposive and snowball sampling techniques, and the process started once the field researcher (GAA) contacted the principal of the special school for CWD and leaders of the rehabilitation centers for CWD to help distribute the study information sheet to potential participants. The information sheet was then posted on the information board at the school and the rehabilitation centers. Initial participants who contacted to confirm their participation were recruited for an interview. They were also asked for help to disseminate the study information sheet to their eligible friends and colleagues who might be willing to take part in the study. This process was carried on, and the recruitment stopped once the researchers felt that the collected information was rich enough to explain the topic being studied and data saturation had been reached, which was indicated in the similarities of information and responses of the last few participants to those of previous ones. Although the recruitment of initial participants was done through both the special school and the rehabilitation centers, most participants had never accessed services provided in these rehabilitation centers for their CWD due to a range of reasons, which will be presented in the results section. None of the potential participants who had stated their willingness to participate withdrew their participation prior to or during the interviews.

Data collection was conducted using one-on-one, face-to-face interviews. Interviews were conducted at a participant-researcher mutually agreed upon time in a private room at the special school for CWD or rehabilitation centers. All participants said that they were comfortable being interviewed in these locations, and this enabled the participants to talk and share their perspectives, views, and experiences comfortably. The interviews were carried out in Bahasa, the first language of the researcher (GAA, male) and participants, and only the researcher and each participant were present in the interview room. The researcher has attended a formal training in qualitative methods during his master’s study and is a freelance researcher. Each interview lasted between 30 to 45 min. Interviews focused on several key areas (drawn from the access to healthcare framework previously described) to understand the participants’ perspectives, views, and experiences in relation to healthcare and rehabilitation services in Belu and the accessibility of the services for CWD. For example, these included their perspectives and views about whether or not the services are sufficiently available, acceptable, and approachable and meeting the health needs of their CWD; their experiences with access to healthcare and rehabilitation services; their views and expectations towards healthcare professionals and therapists who provided the services; and their perspectives and views about various factors (e.g., economic, social, religious, and personal factors) that may have hindered or influenced access to the services. Interviews with the two service providers focused on similar areas although from the perspective of their experiences of providing services; their views on the accessibility, approachability, and acceptability of services they provide; and their thoughts on any barriers that they perceive to services for CWD. No repeated interviews were conducted with any of the participants. There was no established relationship between the researcher and any of the participants prior the interview. At the end of the interviews, each participant was offered the opportunity to read and correct the recorded information after the transcription, but none took the opportunity.

### 2.4. Data Analysis

All interviews were digitally recorded and transcribed verbatim into coding sheets prior to the analysis by the first two authors (GAA and NKF). Transcriptions were imported in NVivo 12 software (version 12, QSR International Pty Ltd, Melbourne, Victoria, Australia) program to facilitate further analysis. Analysis was conducted in Bahasa Indonesia, and relevant quotes were translated into English for this paper. This approach was optimal for two key reasons. Firstly, the interviewer (GAA) is Indonesian and speaks Bahasa as his first language. It was therefore more credible to interpret and code the data using the same language as the research participants. This aligns with the qualitative epistemological position of interpretivism used within our study. Secondly, translating interviews into English before analysis carries the inherent risk of losing both semantic and cultural meanings [43] since a number of terms in Bahasa do not have literal translations and need to be understood in their cultural context. Data analysis was guided by the five steps of qualitative data analysis introduced in Ritchie and Spencer’s framework analysis [44]. The first step is familiarisation with the data. We read each individual transcript repeatedly, marking ideas related to the challenges or barriers the participants experienced in their access the services. We also provided comments to the data extracts to search for meaning, patterns and ideas. The second step is identifying a thematic framework. We did this by making judgement and writing down key issues and concepts from participants, which were used to form the thematic framework. The third step is indexing the data. We performed data index by creating open coding to data extracts in each individual transcript and then making a list of these open codes to look for similar or redundant codes and to reduce them into smaller and manageable number. This was done by grouping together codes referring to the same theme to reach a few overarching themes and sub-themes. The fourth step is creating a chart. The chart was created by arranging thematic framework, which enabled us to compare the data within each interview and across the interviews. The fifth step is mapping and interpretating data as a whole. We performed this by revisiting the codes that were made to extract data, reviewing the summary of the data that were charted, and pulling together all the key characteristics of the data, mapping and interpreting them [44,45].

### 2.5. Ethical Consideration

The ethical approval was obtained from Health Research Ethics Committee, Duta Wacana Christian University, Indonesia. Prior to the interview, the participants were informed that their participation was voluntary, and they could withdraw from the study at any time without any consequences. Participants were also informed about the confidentiality of the information they provided. Anonymity was maintained by using identification letter and number (e.g., R1, R2) for de-identification purposes. Participants were also informed about the duration of the interview (30–45 min) and that the interviews would be recorded using tape recorder, and notes would be taken during interview if necessary. Each participant signed and returned a written consent form on the interview day.

## 3. Results

### 3.1. Sociodemographic Profile of the Participants

A total of 22 mothers or female caregivers and two staff of the two disability rehabilitation centers were involved in this study. The age of mothers or female caregivers ranged between 35–60 years old. The majority of them were married and biological mothers of the children, two women were caregivers (grandmother and aunty), and four women were single parents. Among these mother or female caregivers, the majority (14) graduated from junior high school, six graduated from senior high school, and two finished elementary school. Most of the participants were unemployed (18), two were household assistants (domestic work), and two were shopkeepers. The majority of participants were housewives (18—and four were unmarried or widowed) and from poor families, as husbands generally earned very low income and worked as carpenters, street vendors, vegetable or chicken merchants, or farmers. Thirteen participants were Catholic, six were of a Christian denomination, and three were Muslim. All the CWD (*n* = 22) attended the special school CWD and lived at home with their parents or caregivers. Five participants reported having accessed the services at the rehabilitation centers for their CWD a few times in the past. All the participants acknowledged that they accessed general health care services at hospitals for their CWD only when their children were unwell. Characteristics of the children are provided in Table 1.

### 3.2. Financial Barriers

High costs of healthcare services and poor financial situation, reflected in their inability to afford the costs for the services or treatment, were the main barriers for mothers or female caregivers of CWD in accessing medical treatment or services. The stories of all the participants showed that they experienced financial difficulties due to increased healthcare expenditure for their CWD. The following narratives of two mothers below reflected how poor financial difficulties influenced their decision to stop visiting medical doctors or taking their CWD to therapists:

“I reduced the length of physical therapy from 2 h every three days to 1 h every three days because it was expensive ($12 per hour), and I could not afford it (the mother and her child accessed the therapy in Surabaya, not in rehabilitation services in Belu). Now, you know, I do not take my son (with a disability) to any therapist anymore. I just look after him. I do not want to buy herbal medicines, which are expensive, from Java or Bali”

(R11: 39-year-old mother).

“I usually kept silent anytime the doctor or nurses recommended my son (with a disability) to be taken to Kupang or Java. My mind goes straight to money anytime they said these places. To be honest, my husband and I could not afford travel, accommodation, and to buy recommended medicines. Most of the cases, I stopped visiting the doctor and stopped buying recommended medicines”

(R17: 48-year-old mother).

Inability of mothers or female caregivers of CWD to purchase health insurance was another finance-related barrier to their access to healthcare services for their children. All mothers or female caregivers reported not having health insurance for their CWD due to lack of or low incomes or unemployment. The following story of a mother with two children, of whom one had a disability, clearly reflected the participants’ financial incapacity to afford healthcare insurance for their CWD, which was a barrier to their access to healthcare services:

“Having private insurance means we have to pay every month. To be honest, we cannot afford it, as my husband’s income is for daily expenses, and I am just a housewife with no income. One of my neighbours told me that the monthly health insurance fee of BPJS (Health Care and Social Security Agency) is approximately IDR 5000–100,000 ($4–$8,) which sounds cheap, but it is still expensive for my family. Anytime people suggest me to have health insurance for my son (with a disability), I said it is expensive for us. We are four in this house, and how can we afford?”

(R5: 42-year-old mother).

A similar story was also shared by a 52-year-old grandmother who was a caregiver of her granddaughter with a disability:

“I was once told by the doctor about BPJS (health insurance), but I could not afford the monthly fee, so I do not apply for it. I do not have income, that is why I hardly bring my granddaughter to hospital for medical check-up”

(R1: 59-year-old grandmother caregiver).

Struggling to fulfil daily basic necessities (e.g., food, water, housing) was also a reflection of the financial hardship facing mothers or female caregivers, which was acknowledged to influence their access to healthcare services for their CWD. The majority of the participants reported that they experienced difficulties in fulfilling daily needs, which seemed to lead to the decision to prioritise the fulfilment of basic necessities over other aspects such as healthcare or health insurance:

“I never think about insurance. It is too expensive for us. I am a single mother of three kids; one of them has specific condition (living with a disability). I do not know where the kids’ father is. I do not regularly access healthcare services for my child (with a disability) because I do not have enough money. I cannot afford health insurance either, even just for the one with a disability. I work in the kitchen in a hotel. Sometimes I am a cleaner in the hotel. If I need to buy medicines, I ask for help from my boss, the owner of the hotel”

(R17: 48-year-old mother).

“We (the woman and her husband) do not have monthly salary, and therefore, we do not have health insurance or do regular medical check-up and treatment for my child (with a disability). My husband’s salary as a carpenter is about IDR 100,000 to 150,000 (±$10 to 15) per day. But, you know, it depends on the calling (casual work) from his supervisor. It is not enough for daily living expenses. If the doctor or nurses said that certain medicines require insurance to be able to access them for free, then I just go back home”

(R1: 46-year-old mother).

The rehabilitation staff also echoed the same stories about the poor financial situation of families caring for CWD as a significant issue related to their poor access to healthcare services. The narrative of a female staff at one of the rehabilitation centers as shown below illustrated such assertions:

“I know for sure that most parents or caregivers of children with a disability have low economic status. Most of them do not have permanent income and rely on casual work, which is uncertain, sometimes available, sometimes not. So, things like medical treatments or therapies and health insurance for their children with specific needs seem not the number one priority for many because they are struggling with daily basic needs. The children (with a disability) that we are taking care of in this rehabilitation centre are all from poor families who cannot take care of them properly”

(staff member at a disability rehabilitation centre).

### 3.3. Religious or Faith-Related Factors

#### 3.3.1. Converting Religion Due to Marriage and Being a Non-Catholic

A few participants revealed that they did not access services or therapies provided by the Catholic disability rehabilitation centers run by the Catholic nuns due to some religious reasons. For example, some women described that they converted from Catholic to the religion of their husband after marriage, which made them feel reluctant and embarrassed to access therapies at the Catholic rehabilitation centers. Such feelings were illustrated in the following stories of two mothers who converted their religion to Islam and Protestant:

“Previously, I was a Catholic, but then I converted to Muslim because my husband is a Muslim. I know quite well about the rehabilitation centre for children with a disability organised by Catholic nuns. However, you know, I am reluctant and feel embarrassed to take my son there because I am afraid if they ask what my religion is”

(R8: 43-year-old mother).

“I know religion is not a matter for accessing rehabilitation services. I feel embarrassed to access because I have converted my religion due to marriage, and I follow my husband’s religion. My son is living with a disability, and I am supposed to take him to Catholic rehabilitation centre, but I am concerned with how people might think of me. I was afraid of neighbour’s cynical attitudes”

(R13: 47-year-old mother).

Furthermore, having a religion other than Catholic seemed to also influence some non-Catholic participants’ access to services provided by the Catholic rehabilitation centers for CWD. This was due to their assumption that the services were only for Catholics, which indicated that the participants were not aware of the inclusive services of the Catholic disability rehabilitation centers. The following story of a 47-year-old mother of a son with a disability illustrated this:

“The majority population here is Catholic, and I know there are rehabilitation centers for children with a disability managed by Catholic nuns. I am a Christian (Protestant denomination), I never visited those rehabilitation centers because I think they just serve Catholic people”

(R13: 47-year-old mother).

#### 3.3.2. Unacceptability of Services for Religious Reasons

Unacceptability of services provided at the Catholic rehabilitation centers due to religious-related factors was another barrier to the accessibility of the service among some participants. Some women who converted from Catholic to other religions described that the decision not to take CWD to the Catholic rehabilitation centers for treatment or therapy was influenced by their husband. This was due to their husbands’ concerns about the possibility of their children converting to the Catholic religion if they regularly visited and were treated at those rehabilitation centers. Thus, the women acknowledged that taking their CWD to any Catholic rehabilitation centers was a sensitive issue:

“One day, I talked about the Catholic rehabilitation centers to my husband before we decided to take my son to Surabaya. He (the husband) looked unhappy. I could see from his face that I knew he did not want”

(R8: 43-year-old mother).

“It is not easy to convince my husband to take my son (with a disability) to any Catholic rehabilitation centers. He (her husband) thinks if I often visit the rehabilitation centers, my son will become a Catholic”

(R19: 45-year-old mother).

This issue of religious reasons underlying unwillingness of non-Catholic residents to access rehabilitation services provided in the Catholic nuns at the rehabilitation centers was also acknowledged by the rehabilitation staff interviewed. They described that one of the activities that they had with the children was Catholic prayer, which might be a concern for non-Catholic parents or families to send their CWD to the rehabilitation centers as explained in the following narrative:

“It is clear that non-Catholic parents do not send their children (CWD) to us (to be taken care of at the rehabilitation centre). I think the main reasons are because this rehabilitation centre is run by Catholic nuns, and here, we also teach the children how to pray and pray together as parts of our daily activities. Non-Catholic parents may be concerned with these things, which makes sense…”

(a staff member at a disability rehabilitation centre).

#### 3.3.3. Beliefs in God’s Will

Some participant’s perception about disability condition of their children as “God’s will” also seemed to be an influencing factor for their lack of access to healthcare services for their children. These participants said that intervening against “God’s will” in order to try to make their child “better” would go against their religious beliefs. The story of a mother of a son with a disability, who decided to ignore medical advice from healthcare professionals for her child, illustrated such perception:

“My son has cognitive problem. That’s what the doctor said to me. I do not think my son needs therapy to help him be smarter than he is at the moment. I am happy as long as he is healthy. I do not want to force him to be smart. I believe maybe God wants him to be like he is today”

(R16: 49-year-old mother).

A similar story was echoed by a mother of a daughter with a disability, who believed that her child’s disability was part of God’s plan for the child and the family. Such belief seemed to have an influence on her health-seeking behaviour or access to healthcare services for her daughter:

“I believe that everything happens for a reason and God’s permission. My faith tells me that God has a plan for everybody, and I believe God has a plan for my daughter and our family through her condition. So, I do not want to think too much about treatment or therapy for her condition…”

(R20, 39-year-old mother).

### 3.4. Structural or System Related Barriers

#### 3.4.1. Shortage of Staff at Rehabilitation Centers and Lack of Trust

Structural or system related barriers were also reported to influence mother or female caregivers’ decisions to send their CWD to the rehabilitation centers to be treated. For example, several participants described that the shortage or limited number of staff and lack of trained staff to deliver services for CWD were the underlying reasons for them not sending their children to those rehabilitation centers:

“I know from my neighbours that the rehabilitation centers for children with a disability are free. However, the Catholic nuns serve and care for many children with a disability. If I took my daughter (with a disability) there, it might add more workload for the nuns”

(R4: 50-year-old mother).

“I once asked people (volunteer workers) at the disability rehabilitation centers, and I was told that the rehabilitation centers find it difficult to get people to be trained as therapists”

(R15: 52-year-old mother).

Building on perceptions that some staff are not appropriately trained, some participants then talked about their lack of trust in the skills of staff at rehabilitation centers, which was a key reason for them not to utilise services provided at those centers. This led to the concerns about the safety of their children and the likelihood of sub-optimal or even unsafe therapy, which might endanger the life of their children. The following quotes from the participants reflected such concerns:

“I doubt the skills of the therapists in the rehabilitation centers here. I watched on TV many children (with a disability) died due to malpractice therapy. I do not want my son (with a disability) getting the same experience; otherwise, his condition might get even worse”

(R15: 52-year-old mother).

“I know the disability rehabilitation centers have a sincere heart to help children with a disability. However, to be a therapist, someone needs to have a certified training. If they do not have a certificate that allows them to do the therapy, then I still doubt, and I do not put my daughter at risk”

(R7: 49-year-old mother).

Shortage of staff was also acknowledged by the rehabilitation staff interviewed, as presented in the following quote:

“We are taking care of tens of children in this rehabilitation centre, and for sure, we have very limited staff. We always try to do our best with very limited available resources to help the children because this is our mission. A few nuns here dedicate themselves to help the children”

(a staff member at a disability rehabilitation centre).

#### 3.4.2. Unavailability of Specialists Trained in Treating Different Types of Disabilities

Unavailability of disability specialists was also a barrier for mothers or female caregivers to accessing healthcare services for their CWD. Several mothers reported that they never talked to any service providers or medical doctors who were specialised in the disabilities experienced by their children. The belief by participants was that such highly trained professionals were not available in Belu. As a consequence, they did not regularly access healthcare services for their CWD:

“I once asked the doctor whether there is any specialist in autism in our district, and the doctor said no. He (general practitioner) just recommended me to go to Java to do therapy for my child with a disability”

(R17: 48-year-old mother).

“My nephew has walking difficulty. I never take him to a specialist because I think there is no specialist here. There are many nurses or health workers but not for disability issues. Here, having a child with a disability means you have to help yourself, as there are no healthcare professionals or health workers who are specialised in any disability conditions”

(R2: 48-year-old female caregiver/aunty).

### 3.5. Low Perceived Need for Health Care or Treatment for CWD

Low perceived need for healthcare or rehabilitation services was also indicated as a barrier to the accessibility of the services for CWD. Several participants described that their CWD did not need health treatment or therapy from healthcare professionals and therapists, which seemed to influence their decision to not access the available services for their children. Such decision seemed to also be influenced by a lack of perceived benefits of the services for their children:

“My son has a problem with thinking, attention, and memory. He does not need a teacher or a therapist to teach or train him be good academically”

(R16: 49-year-old mother).

“My neighbours encouraged me to take my daughter who cannot speak to therapists, but I said no because what my daughter needs is understanding. I and also other people around her just need to understand her. That is what she needs, and I do not think she needs therapists to be able to speak”

(R3: 43-year-old mother).

Other mothers reported feeling less convinced after visiting general practitioners or nurses. This was acknowledged as making them feel bored and as if it is unnecessary to access the services of these healthcare professionals for their CWD, as illustrated in the following narrative from a mother of son with a disability:

“Sometimes my husband and I felt bored whenever we visited general practitioners. My son has a hearing impairment. I think general practitioners treat my child like a normal child. But anyway, we cannot do anything. We cannot expect more than that, as there is no specialist in our district. Sometimes, I feel that it is useless to consult general practitioners or nurses”

(R11: 39-year-old mother).

However, it also seemed that less approachability of services at the rehabilitation centers reflected the lack of dissemination of information about the services within communities in Belu, and this also contributed to the participants’ low perceived need for the services for their children. This was indicated in the following quotes from the participants:

“As I said before, I do not know much about the services at the rehabilitation centers of the Catholic nuns. I heard information about these rehabilitation centers, but it is just mouth-to-mouth information from friends and families because they know that my child has special needs. I do not hear it from the nuns; that is why, like I said earlier, I do not even know that they also serve kids who are not Catholic”

(R13: 47-year-old mother).

“We do not carry out seminars or information sessions for communities; I am not sure about the reasons why. I guess budget and limited number of staff we have might also be the reasons… I am not sure whether there are people who do not know about the services provided by this congregation”

(a staff member at a disability rehabilitation centre).

## 4. Discussion

Accessibility to healthcare and rehabilitation services is an essential element to improving health and wellbeing of people with a disability, including children. This paper has explored the key barriers to the access to healthcare and rehabilitation services for CWD in Belu district, Indonesia. The findings suggest that high costs and inability to pay the costs of the healthcare services and health insurance were the main financial barriers to the access of mothers or female caregivers to healthcare services for their CWD. The participants’ poor financial situation due to unemployment, unstable jobs, and low or lack of incomes, which led them to prioritising the fulfilment of daily basic necessities over health care or treatment for their CWD, was the main reason for their inability to afford the costs of healthcare services. The findings are consistent with previous research findings reporting financial constraints reflected in the inability of parents or caregivers to afford costs for medical treatment, health insurance fees, and social protection as a barrier to the access to healthcare services or treatment for CWD [4,5,6,10,11]. The findings are also in line with the access to healthcare framework’s concepts of affordability and ability to pay, which suggest high prices of healthcare services and economic incapacity of people with a health-related need to pay for the services as barriers to the accessibility to healthcare services [31,33].

The current study also reports new findings that have not been explored in previous studies [21,35,46]. For example, it suggests that religious or faith-related factors, such as being a non-Catholic or converting from Catholic to another religion after marriage, influenced participants’ health-seeking behaviours and acted as barriers to their access to rehabilitation services provided at Catholic disability rehabilitation centers. Such factors led to the participants feeling reluctant, scared, and/or embarrassed and afraid of the possibility of negative reactions or cynical attitudes from others. The influence of these factors was also supported by the participants’ assumptions that the services provided at the rehabilitation centers were only for children who are Catholic. The findings seem to also indicate less dissemination of information about services at the rehabilitation centers to community members or families in need, which supports the construct of the access to healthcare framework about approachability of healthcare services as a dimension that determines accessibility to the services by people with a health-related need [31,33]. The current findings suggest that religious or faith-related factors were also the underlying reasons for unacceptability of the services provided at the rehabilitation centers by some non-Catholic participants’ husbands, which influenced their access to the services for their CWD. Such unacceptability seemed to be influenced by their husbands’ concerns and assumptions about the possibility of their children converting to Catholic religion in the future. This conforms to the concept in the access to healthcare framework, which suggests that acceptability of healthcare services is determined by whether or not the services are provided or delivered in ways that are socially, culturally, and religiously acceptable [31,33]. The participants’ religious beliefs that disability condition of their children is “God’s will and plan”, were also barriers to their access to healthcare services, for such beliefs led to their perceptions that seeking healthcare for their CWD was a waste of effort and did not conform to their religious beliefs.

Consistent with the findings of previous studies [22,23,24], the current study suggests that structural barriers, such as shortage of staff at rehabilitation centers and lack of trust in the skills of staff providing therapy services, influenced the participants’ access to the rehabilitation services. It is plausible to speculate that shortage of staff at the rehabilitation centers and distrust in the staff’s therapy skills may have led to the participants’ assumption of the possibility of therapy malpractice, which could worsen the condition of their children. Similarly, the current study reports the unavailability of medical doctors who were specialists in any disability conditions as an influencing factor for the participants’ access to healthcare services for CWD. The findings are in line with the findings of previous studies [10,21,47] and the concept in the access to healthcare framework that suggests that accessibility to healthcare services is determined or influenced by whether or not qualified healthcare professionals are available to provide the services [31,33].

The current findings suggest that participants’ low perceived benefits of the healthcare and rehabilitation services for their CWD was a barrier to their access to the services. This was influenced by dissatisfactory experiences of healthcare services and less convincing medical advice the participants received from general practitioners or nurses and the lack of dissemination of information about services provided at the rehabilitation centers. These are in line with the access to healthcare framework’s concept about ability to perceive need for care as a determinant for the accessibility to healthcare services [31]. The findings seem to reflect the participants’ poor knowledge and information about how their children could benefit from the available services, which are in line with previous findings reporting low health literacy as a barrier the accessibility to healthcare services [33,48] but also suggest a need for healthcare services to disseminate appropriate information about their services to families.

### Study Limitations and Strengths

There are several limitations that need consideration when interpreting these findings. Firstly, like many case studies in qualitative research, the findings of the study may reflect the unique conditions of the participants in Belu district, which may be different to participants caring for CWD in different settings with different characteristics. Secondly, we only explored the perspectives of female caregivers at one point in time and did not explore views of other family members. This may have led to incomplete overview of barriers to the access to healthcare and rehabilitation services although it seems that the mothers and female caregivers were the primary caregivers for the CWD and therefore had the most experience of barriers to healthcare services. However, to our knowledge, this study represents an initial qualitative understanding of barriers for mothers or female caregivers to accessing healthcare and rehabilitation services for their CWD in the context of Indonesia and the Belu district in particular. Thus, the current findings are useful to inform disability-related programs and interventions to address the needs of CWD in Belu and other similar settings in Indonesia and globally. Future studies on this topic that involve a significant number of mothers and fathers, other family members, and caregivers of CWD are recommended, as they may provide a more complete picture of barriers to the access to supports and services.

## 5. Conclusions

The study reports several barriers to the access of mothers or female caregivers to healthcare and rehabilitation services for CWD. These barriers were financial, religious, or faith-related factors; structural or system-related barriers; and low perceived need for care for their CWD. The findings highlight a critical need for public disability rehabilitation centers managed by the government to support public access to rehabilitation services. The findings also indicate the need for the provision of qualified therapists and healthcare professionals who are specialists in disability conditions to serve CWD. As costs of healthcare services and poor financial situation are amongst the main barriers to the access to healthcare and rehabilitation services for CWD, interventions that address economic aspects of mothers or female caregivers and families caring for CWD in Belu and other similar settings in Indonesia are recommended. Provision of fully subsidised healthcare insurance and financial capital aid for small-scale business for parents of CWD would represent examples of interventions helpful in caring for CWD and their families.

## Figures and Tables

**Table 1:** Characteristics of the children.

Type of Disability	Number of Children	Age Range (Years)
Visual impairment	3	8–13
Hearing impairment	3	9–15
Speech impairment	4	6–14
Physical handicap	5	6–14
Cognitive impairment	3	10–15
Mental and physical impairment	4	10–16

## Data Availability

The data presented in this study are available on request from the corresponding author. The data are not publicly available due to restrictions set by the human research ethics committee.

## Supplementary Materials

The following are available online at https://www.mdpi.com/article/10.3390/ijerph182111546/s1, Figure S1: COREQ Checklist.
