# Supplementary material for: Understanding Barriers to the Access to Healthcare and Rehabilitation Services: A Qualitative Study with Mothers or Female Caregivers of Children with a Disability in Indonesia
Source: Int J Environ Res Public Health. 2021 Nov 3;18(21):11546. doi: 10.3390/ijerph182111546 (PMC8583444; doi:10.3390/ijerph182111546)
Supplement: Supplementary file 1 [file ijerph-18-11546-s001.zip › ijerph-1418220-supplementary.pdf]

Table 1 Consolidated criteria for reporting qualitative studies (COREQ): 32-item checklist

No	Item	Guide questions/description	Page
**Domain 1: Research team and reflexivity**			
Personal Characteristics			
1.	Interviewer/facilitator	Which author/s conducted the interview or focus group?	4
2	Credentials	What were the researcher's credentials? E.g. PhD, MD	4
3	Occupation	What was their occupation at the time of the study?	4
4	Gender	Was the researcher male or female?	4
5	Experience and training	What experience or training did the researcher have?	4
Relationship with participants			
6	Relationship established	Was a relationship established prior to study commencement?	4
7	Participant knowledge of the interviewer	What did the participants know about the researcher? e.g. personal goals, reasons for doing the research	4
8	Interviewer characteristics	What characteristics were reported about the interviewer/facilitator? e.g. Bias, assumptions, reasons and interests in the research topic	4
**Domain 2: study design**			
Theoretical framework			
9	Methodological orientation and Theory	What methodological orientation was stated to underpin the study? e.g. grounded theory, discourse analysis, ethnography, phenomenology, content analysis	2-3
Participant selection			
10	Sampling	How were participants selected? e.g. purposive, convenience, consecutive, snowball	4
11	Method of approach	How were participants approached? e.g. face-to-face, telephone, mail, email	4
12	Sample size	How many participants were in the study?	4
13	Non-participation	How many people refused to participate or dropped out? Reasons?	4
Setting			
14	Setting of data collection	Where was the data collected? e.g. home, clinic, workplace	4
15	Presence of non-participants	Was anyone else present besides the participants and researchers?	4
16	Description of sample	What are the important characteristics of the sample? e.g. demographic data, date	5-6
Data collection			
17	Interview guide	Were questions, prompts, guides provided by the authors? Was it pilot tested?	4
18	Repeat interviews	Were repeat interviews carried out? If yes, how many?	4
19	Audio/visual recording	Did the research use audio or visual recording to collect the data?	5
20	Field notes	Were field notes made during and/or after the interview or focus group?	5
21	Duration	What was the duration of the interviews or focus group?	4
22	Data saturation	Was data saturation discussed?	
23	Transcripts returned	Were transcripts returned to participants for comment and/or correction?	4

**Domain 3: analysis and findings**			
Data analysis			
24	Number of data coders	How many data coders coded the data?	4
25	Description of the coding tree	Did authors provide a description of the coding tree?	6-12
26	Derivation of themes	Were themes identified in advance or derived from the data?	5
27	Software	What software, if applicable, was used to manage the data?	4
28	Participant checking	Did participants provide feedback on the findings?	4
Reporting			
29	Quotations presented	Were participant quotations presented to illustrate the themes / findings? Was each quotation identified? e.g. participant number	6-12
30	Data and findings consistent	Was there consistency between the data presented and the findings?	6-12
31	Clarity of major themes	Were major themes clearly presented in the findings?	6-12
32	Clarity of minor themes	Is there a description of diverse cases or discussion of minor themes?	12-14
